# Improved Pose Estimation of Aruco Tags Using a Novel 3D Placement Strategy

**DOI:** 10.3390/s20174825

**Published:** 2020-08-26

**Authors:** Petr Oščádal, Dominik Heczko, Aleš Vysocký, Jakub Mlotek, Petr Novák, Ivan Virgala, Marek Sukop, Zdenko Bobovský

**Affiliations:** 1 Department of Robotics, Faculty of Mechanical Engineering, VSB-TU Ostrava, 70833 Ostrava, Czech Republic; petr.oscadal@vsb.cz (P.O.); dominik.heczko@vsb.cz (D.H.); ales.vysocky@vsb.cz (A.V.); jakub.mlotek@vsb.cz (J.M.); petr.novak@vsb.cz (P.N.); 2 Department of Mechatronics, Faculty of Mechanical Engineering, Technical University of Košice, 04200 Košice, Slovakia; Ivan.Virgala@tuke.sk; 3 Department of Robotics, Faculty of Mechanical Engineering, Technical University of Košice, 04200 Košice, Slovakia; marek.sukop@tuke.sk

**Keywords:** Aruco, pose estimation, 3D grid board, precision

## Abstract

This paper extends the topic of monocular pose estimation of an object using Aruco tags imaged by RGB cameras. The accuracy of the Open CV Camera calibration and Aruco pose estimation pipelines is tested in detail by performing standardized tests with multiple Intel Realsense D435 Cameras. Analyzing the results led to a way to significantly improve the performance of Aruco tag localization which involved designing a 3D Aruco board, which is a set of Aruco tags placed at an angle to each other, and developing a library to combine the pose data from the individual tags for both higher accuracy and stability.

## 1. Introduction

In robotics, engineers attempt to get the maximum usage out of the available sensors, often outside the recommended use cases. One popular example would be the use of monocular vision to get a rough estimate of the 3D world being imaged, which is done by using mathematical models like the pinhole camera model [1,2,3,4,5]. However, to make use of the pinhole camera model, we have to experimentally determine the characteristics of the camera—the distortion coefficients (radial and tangential distortion), as well as the camera matrix, the accuracy of which greatly relates to the accuracy of the 3D world reconstruction that we get. This paper starts with this calibration process, which was found out to be improvable using a straightforward approach. Some of these approaches to calibrate the cameras have been discussed in the literature. In [6], the authors used a single image to calibrate the camera online while accepting a low calibration accuracy. The Camera Calibration Toolbox integrated in MATLAB was used and demonstrated in [7]. The methods in [6,7] provide calibration to be obtained quickly, but with lower accuracy. Another study [8] presented a calibration method that uses the transformation of the shape of a circle from the world plane to the image plane to determine the internal parameters of the camera. Based on a comparison of four methods, this approach proposes good effectiveness but assumes a high-resolution image in order to keep the properties of the circle. Grids at different depths (3D points) were used to calibrate the stereo-vision system in [9]. Article [10] dealt with camera calibration and reuses this calibration for different camera modes. The study in [11] was based on marker detection, where the detected position of the robot was compared between the position estimates from internal and external cameras. It is also possible to utilize genetic algorithms [12] for the calibration. The use of Aruco tags, made available by the Open CV library [13], was used to help estimate the pose of objects in images taken by a single camera from a single perspective. The OpenCV Aruco module provides the users with a simple interface to plug in the characteristics of the tag they desire to detect the pose. It takes as input a 2D image containing the tag along with the camera parameters to give out an estimate of the pose of the tag in a 3D coordinate space relative to the camera. An alternative to Aruco provided by OpenCV is Charuco, which embeds Aruco-like tags in a chessboard. Charuco is more accurate than the Aruco grid, thanks to the included chessboard and takes over advantages of tag-based calibration which supports occluded or partially visible calibration patterns. However, there are many other types of calibration patterns, such as circle-based patterns, Halcon, Kalibr [14], CALTag [15] and others mentioned in [16]. Another method mentioned in [17] uses only a modified chessboard to calibrate cameras. This system works by recalibrating the camera parameters when it is necessary to detect the camera position and orientation, which has both advantages and disadvantages if only the camera position changes.

## 2. Calibrating the Camera

**Figure 1:**
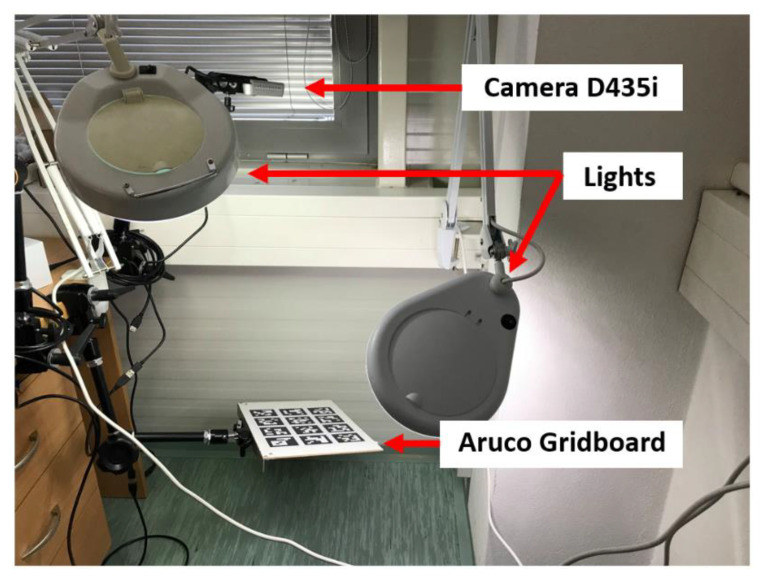
Calibration process.

In order to localize the Aruco tags precisely, we need to know the calibration matrix of the camera in use [18]. OpenCV provides a systematic pipeline to do exactly so [19]; however, getting the camera’s intrinsic characteristics is a process prone to approximation errors, and it generally requires several datapoints to get right. A bad calibration results in an incorrect stereographic projection, which in this case would lead to bad pose estimates of the tag. Through this paper, a set of Intel RealSense D435i cameras were used to eliminate errors coming from any particular device. Our attempts to calibrate the camera using the standard procedure were initially amiss during several attempts to do so. Constant illumination was maintained throughout the process, and various orientations of the calibration board were imaged to maximize the number of points available for more precise calibration. The experimental setup is shown in Figure 1.

This calibration process yielded the parameters shown in Equations (1) and (2).

(1) $$camera^{\prime }s\;distortion\;Coefficients=\left(\begin{array}{ccccc} 2.1479 & 46.0661 & 0.0708 & -0.1847 & 0.8319 \end{array}\right)$$

(2) $$camera^{\prime }s\;intrinsic\;parameters=\;\left(\begin{array}{ccc} 5577.3914 & 0 & 362.06\\ 0 & 5884.5847 & 649.0561\\ 0 & 0 & 1 \end{array}\right)$$

**Figure 2:**
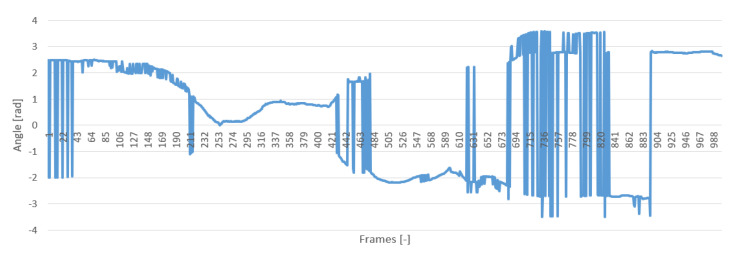
Roll angle during manual measurement.

The intrinsic parameters of the camera and its distortion coefficients describe the nature of the projection of the imaged objects from the world to the image plane [19]. To test the parameters, we used it to detect the pose of an Aruco tag while rotating the tag slightly along its X-axis (Roll). The camera resolution for this test was set to 1280 × 720, and the frames were recorded at 30 frames per second (FPS). Figure 2 shows the results of this test. Notice the jumps in the detected angle.

The results indicate that the calibration matrix was not accurate; however, the experiment was repeated several times, and the reprojection loss with these parameters was calculated to be close to 2 × 10^−14^. While calibrating, the OpenCV guideline for calibration was strictly followed with 30 images being input into the calibration function with different positions and orientations in the field of view (FOV) of the camera.

## 3. Benchmark for Pose Estimates

**Figure 3:**
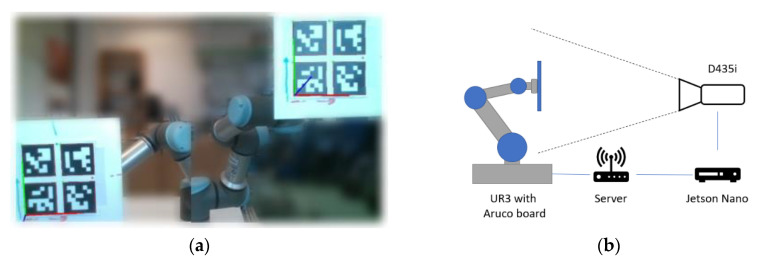
Workplace: (**a**) two measurements at the edge of the measurement range; (**b**) general block diagram.

To know how accurate the Aruco Tag detection was with the currently acquired parameters, a test was devised which involved moving the Aruco tags in the camera FOV along precisely defined positions in the 3D space (Figure 3a shows two such positions). A workplace was created for this evaluation which allowed us to perform automated and precise measurements using a UR3 collaborative robot. A flat grid with 4 Aruco tags was fixed at the TCP (tool centre point) of the robot, and the camera used in the benchmark (Intel RealSense D435i) was fixed in the robot coordinate system. RealSense D435i cameras come with an inbuilt inertial measurement unit (IMU) which we used to ensure that the camera was pointing in the right direction parallel to the TCP Z-axis. To ensure that the distance between the camera and the tag remained constant while performing the benchmark, we used data from the depth stream to align them before the benchmark. The camera was configured to grab frames at a resolution of 1280 × 720 and a framerate of 30 FPS; the data from this camera were passed onto a Jetson Nano, which performed the pose estimation and passed the data to the server (Figure 3b). The server compared these data with the expected data to detect the amount of error in the detection of the Aruco tag at that particular pose in the frame. The library to control a robot from Universal Robots named URX [20] was used for communication with the real world robot.

Table 1 lists information about the setup of the Aruco grid.

All of the coordinate frames (of the individual tags in the camera coordinate frame) were transformed relative to the robot coordinate system. In order to cover the entire FOV of the camera in the experiment, a grid of 30 × 30 positions (for the Aruco tags) was defined with constant spacing between the positions for the detected tag pose in the frame. The benchmark also involved rotating the Aruco board along its X, Y, and Z axes (Roll, Pitch, Yaw) within the range from −30° to 30° with a step size of 15° for each axis to check the impact of angle on pose estimation. Algorithm 1 describes the measurement process.
**Algorithm 1** The Measurement Process.**for***i* = 0 to m   // m[1:30] represents the number horizontal positions for the effector **set_y_position_of_end_effector** (i)  **for**
*j* = 0 to *n* // *n*[1:30] represents the number of measurements in a row   **set_x_position_of_end_effector** (j)  **for**
*k* = 0 to *o* // *o*[1:10] represents the number of frames in the measured position      Grab image      Process image      Save data after processing    **end for**   **end for** **end for**


**Figure 4:**
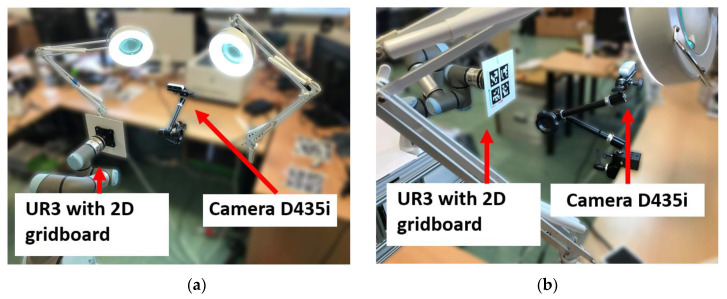
Measuring workplace (**a**) back view; (**b**) front view.

The tagged effector was illuminated to ensure constant lighting of the grid board (Figure 4). The robot gradually moved the tag through the positions according to the defined grid. The measurements were made only when the manipulator stabilized in the desired position for which we added a small delay between reaching the position and the pose being detected. Subsequently, the pose estimation was run ten times for each pose of the tag, and the data were stored to disk. All measurement data were then stored in files for further processing. One measurement took approximately 30 min. All measurements were repeated ten times to collect more data so we could avoid systematic errors.

The range of the effector of the robot limited the range of experimental positions, as it had to be ensured that all the positions were imaged consistently for all the orientations. The orientations that we could perform the measurements for are mentioned in Table 2.

## 4. Benchmark Results for the 2D Aruco Board

**Figure 5:**
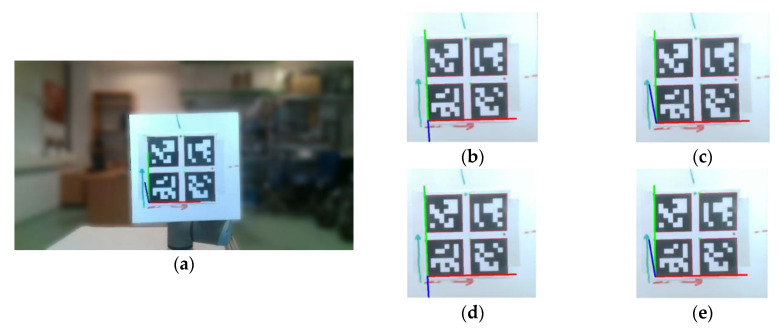
Images from measurements: (**a**) full-frame; (**b**–**e**) observation in four frames consecutively.

During the experiments, one of the more prevalent errors came in the form of unexpected jumps in the approximation of pose in which the detected axes would change by several degrees with a minor change in the actual position of the tag. This problem is recorded and shown in Figure 5, in which a,b,c,d,e are measurements with a minuscule change in the position of the tag, but the detected pose of the blue Z-axis (Yaw) shifts noticeably.

**Figure 6:**
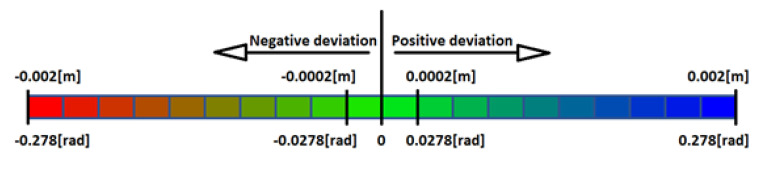
The colour bar represents the range of deviations and the colour to represent it throughout this paper. For angle estimates, the range of deviation is ± 0.278 rad whereas, for the position, the range of deviation is ± 0.002 m.

The experimental results are visualized in the form of a 30 × 30 matrix of deviations, where the deviations are represented with colors determined by the colour bar in Figure 6.

**Figure 7:**
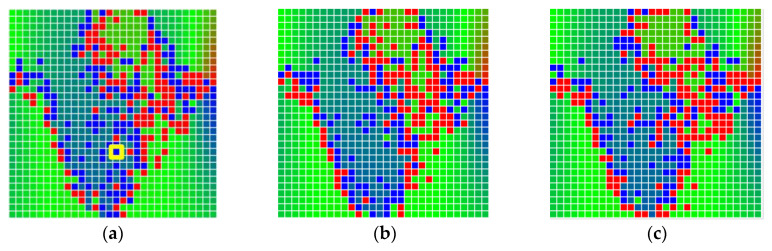
Deviation map observed for Roll estimate of the 2D Aruco grid board: (**a**) measurement 1, observe the highlighted square, this square is for the frame in Figure 5a; (**b**) measurement 5; (**c**) measurement 10.

Each little square in Figure 7 represents a relative position of the Aruco tag in the frame with its neighboring pixels; for example, the deviation for the measurement in Figure 5a would be represented by the highlighted little square approximately in the center of the grid in Figure 7a. The deviation map remained almost consistent across several iterations of the same measurement (for the same angle and distance); an example of this can be observed in Figure 7, where a, b and c are different iterations of error measurements in Roll value of the pose estimate, while the normal of the tag was parallel with the camera Z-axis.

The data from the benchmark with a systematic set of variations of the pose of the tag within the frame are shown in Table 3; it represents data from 10 iterations of the same measurement process. Each row is for a particular orientation of the tag, while the columns depict the benchmark results.

Table 3 leads us to the inference that the deviation map greatly varies with the rotation of the tags. To make the results clear, the deviations observed while detecting the pose of the tag are presented in the form of histograms in Table 4.

**Figure 8:**
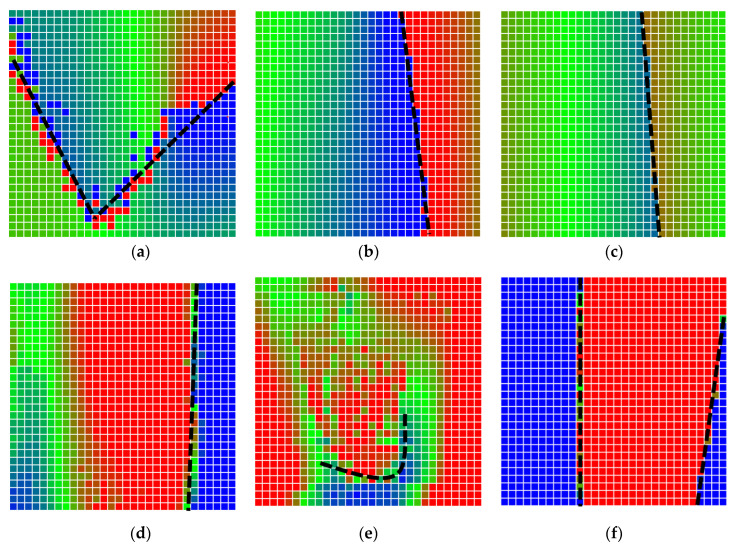
The black lines depict step boundaries; when the tag moves across this line, a sudden change in the detected tag orientation is observed. Observations for (**a**) Roll; (**b**) Pitch; (**c**) Yaw; (**d**) X; (**e**) Y; (**f**) Z.

The error is large while moving along the +Z-axis (Yaw); however, changes in the Yaw angle have small effects on the deviations. This is expected as when the board moves away from the camera, the pose estimate becomes more sensitive to noise and errors in calibration. Rotation of the tag sometimes leads to the formation of a boundary at which moving the tag by a small amount leads to a significant change in the detected orientation. This step boundary is shown in Figure 8 and is similar to what was observed in Figure 5.

## 5. Recalibration with a Liquid-Crystal Display

**Figure 9:**
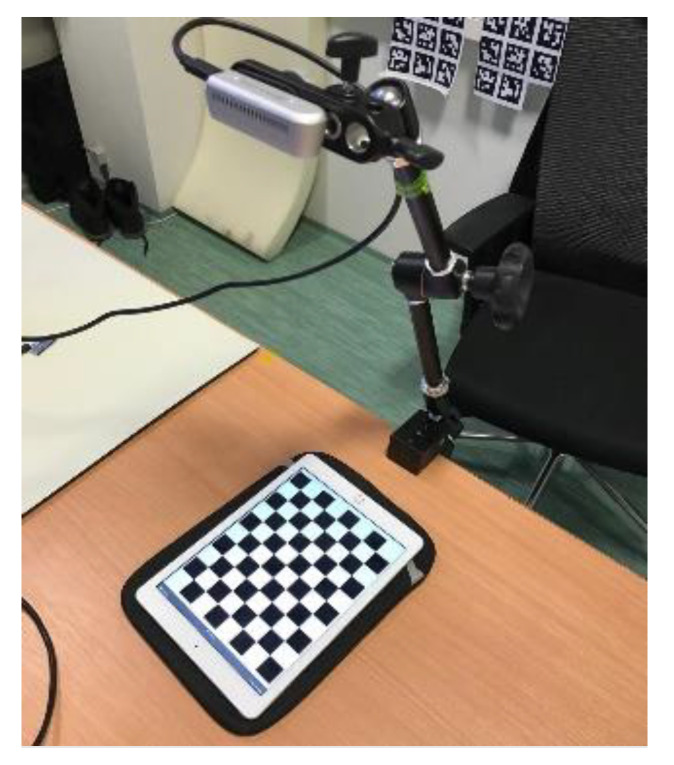
The calibration with a Liquid-crystal display.

The observations from the benchmark process mentioned in Table 3 justified that the standard calibration process yielded a set of parameters that left a lot of instability in the Aruco tag detection and the estimation of its pose. Upon observing the fact that the benchmark results were different for +15° Roll and −15° Roll, although a symmetry was expected, it was decided that a recalibration was required. On the second attempt, instead of printing the calibration board anew, we decided to use a Liquid-crystal display to display the board, as it is never warped (stays completely flat) and is backlit, which should help to detect the board better (see Figure 9). The dimensions of the chessboard squares were measured and were input into the calibration algorithm.

The results from the new calibration are mentioned in Equations (3) and (4).

(3) $$camera^{\prime }s\;distortion\;coefficients=\left(\begin{array}{ccccc} 0.1679 & -0.4901 & -0.0032 & -0.0011 & 0.4101 \end{array}\right)$$

(4) $$camera^{\prime }s\;intrinsic\;parameters=\;\left(\begin{array}{ccc} 902.9293 & 0 & 634.7668\\ 0 & 902.3267 & 354.3479\\ 0 & 0 & 1 \end{array}\right)$$

The effects of the improved calibration are mentioned further in this paper, this calibration will be referred to as “new calibration” and the previous calibration will be called “old calibration”.

## 6. Three-dimensional Aruco Grid Board

The observations made on the 2D Aruco grid-board led to these inferences:

1. Pose estimates are unstable when the orientation of the markers relative to the camera is perpendicular, i.e., the camera Z-axis is overlapping with the marker Z-axis.
2. An error detection metric with redundant tags being detected at the same time can be used to improve pose estimation, i.e., if we have five tags and we know one of them has a pose estimate which is considerably different from the others, we could discard it and use the other tags for pose estimation.

**Figure 10:**
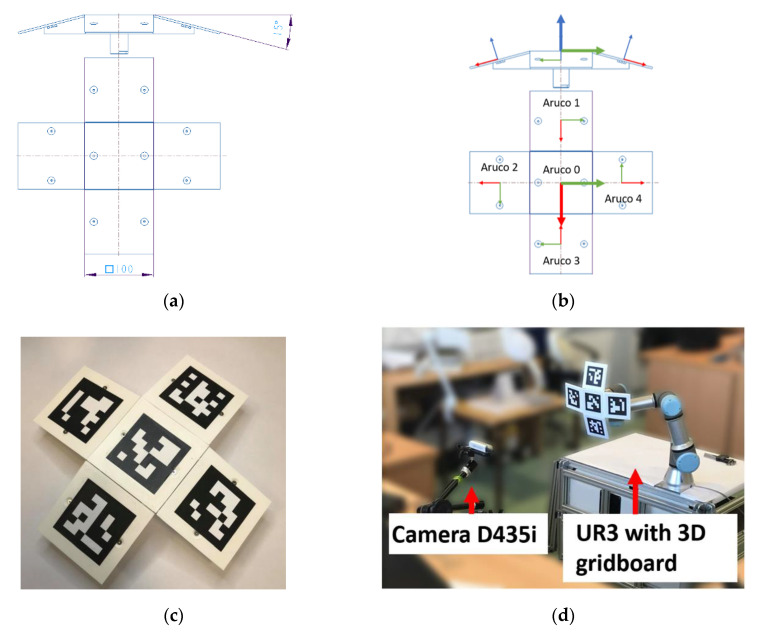
Three-dimensional grid board: (**a**) schema; (**b**) position and orientation of individual tag on the 3D grid board; (**c**) the real 3D grid board; (**d**) the UR3 robot with the 3D grid board.

Given the first inference, it was decided that a 3D organization of the tags should ensure that no two tags are normal to the camera at any point; therefore, a 3D grid board was proposed that consisted of five Aruco tags on five different planes angled to the center plane at 15° (see Figure 10a). This allowed having multiple tags in one image with a known rigid transformation from any tag to the center tag. It also allows the development of a metric to detect the error in the net detected pose (the second inference). The characteristics of the grid are mentioned in Table 5. The grid board was mounted on a UR3 robot (Figure 10d), and the robot coordinate system was used for all the measurements.

The first step of the process was to detect the tags and transform their detected pose from the camera coordinate frame to the robot coordinate system. A Python module was written for this purpose that enables modelling the transforms for such 3D grid boards. For the grid itself, the local coordinate frame is identical with the coordinate system of the center tag.

An example of the rigid transform between a non-central tag (Aruco 1, Figure 10b) to the central tag (Local Coordinate System of the grid board) is depicted in Equation (5).

(5) $$M_{T1\to LCS\;}=\;\left(\begin{array}{cccc} 0.9659 & 0 & -0.2588 & -0.1\\ 0 & 1 & 0 & 0\\ 0.2588 & 0 & 0.9659 & -0.0129\\ 0 & 0 & 0 & 1 \end{array}\right)$$

## 7. Error Metric for the 3D Grid Board

**Figure 11:**
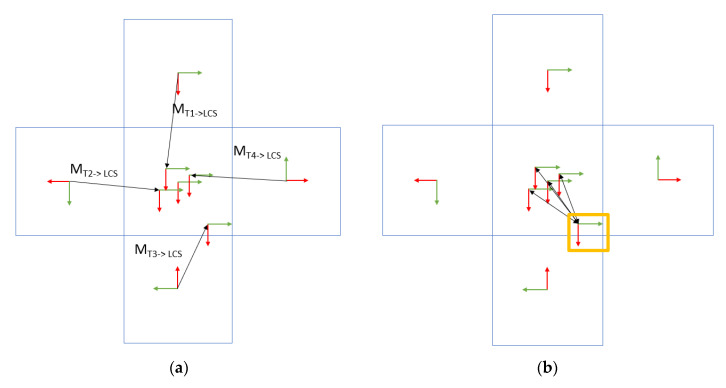
An illustration (not to scale) to describe the error measurements and correction. (**a**) transforms the tag poses from their coordinate frame to the center tag frame. (**b**) shows the error in deviation observed during the experiment, the detected pose marked with the yellow box has the highest deviation.

To ensure that the net pose calculated from the 3D grid board benefits from the redundancy of having five tags with the rigid transformations known between the individual tags, an error metric was devised to detect if any subset of the tags was detected incorrectly. First, all the individual tags were detected, and their pose was estimated, then the detected poses of the non-central tags were transformed to the coordinate frame of the central tag (Figure 11a). Once this was done, the tag with the highest distance (Figure 11b) from the expected position was eliminated from the net pose estimate to improve the accuracy. In order to measure the error in the final pose estimate, we defined an error metric that took as its input the transformed origins of all the non-central tags (and the distance from the real center for the central tag) and computed a scalar that described the error in the net pose estimate. This error metric is described in Algorithm 2.
**Algorithm 2** Calculating the Error in the Net Pose Estimation of the 3D Grid BoardDetect *tvecs* of Tags    //*tvecs* represent array of translations of the origin of detected tags in **for**
*i* = 0 to **length**(*tvecs)                //*camera’s coordinate frame **for**
*j* = 0 to **length**(*tvecs)* *deviation_distance[i]* += (**distance**(*tvecs[i] – tvecs[j]*)) //*calculate distance between transformed*                //*tag centers* **end for** **end for** *filtered_tvecs*
**= remove_detections_with_high_deviation** (*tvecs, deviation_distance*) *volume* = *(***get_max_x***(filtered_tvecs)–***get_min_x**(filtered_tvecs)) *  // get min and get max functions *(***get_max_y***(filtered_tvecs)–***get_min_y**(filtered_tvecs)) *  // return the min and max tvecs      (**get_max_z**(*filtered_tvecs*)–**get_min_z**(*filtered_tvecs*)) // along each axis


## 8. Results

This section discusses the results from experiments across this paper and compares the results between different benchmarks to identify how the different grid boards performed. Table 6 lists the indices used across this section to label the different experiments done.

The benchmark results for all the cases are shown and compared in Table 7, the error metric was used for the 2D grid board as well, and the worst detected tag was filtered from the net pose estimate for all cases mentioned except for M0, which is given for reference (Table 3). M0 is the observation when directly using OpenCV’s grid board pose estimation.

The observations confirm that there was an increase in the accuracy of the detected pose when the proposed 3D grid board was used, and the results are consistent across different measurements. There is still an increase in error as the tag was moved away from the camera (+Z-axis), but that is expected because the tags are more distant from the camera. The histograms in Table 8 confirm the fact that the large deviations from real pose estimates have been significantly reduced in measurements done with the 3D grid board (i.e., most of the deviations are small). For example, in M1 (the first row in Table 8), we observe that there are more data points with highly positive (blue) and negative (red) deviations compared to M4 (the last row), where there are more data points with small deviations (green).

**Figure 12:**
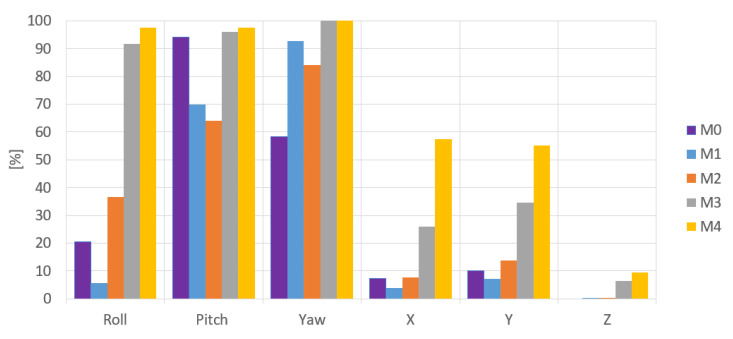
Improvement in pose estimation across different grid boards and camera calibrations.

Figure 12 shows the percentage of small deviations ±5% of the full range (the two center bars from the bar graphs shown in Table 8). The histograms show an increase in small deviations as a larger fraction of all the deviations, which corresponds to a decrease in the number of large deviations observed.

When recording the frequency of deviations, Figure 12 confirms that compared to M0, the accuracy of the pose estimate of M4 increased during Roll by 77%, Pitch by 3%, Yaw by 42%, and along X by 50%, Y by 45% and Z by 9%.

## 9. Conclusions

This paper presented a new way of using Aruco tags to detect the pose of objects in 3D space. To test the pose estimation accuracy and compare different tags’ performance, a standard benchmark was created that tested the error in pose estimates across all parameters. This benchmark was applied over a 2D grid placement of tags, and the observations were used to determine that a 3D organization of multiple tags performs better than a 2D placement of tags. Different visualizations were created to demonstrate the observations appropriately. All experiments were done using Python, where they were first simulated in a simulation environment and then replicated in the real world. UR3 collaborative robot was used to ensure that the measurements were consistent and multiple Intel Realsense D435 cameras were used interchangeably to avoid errors coming from any particular camera. A Python library [21] was written that allowed the implementation and benchmarking of arbitrary 3D organization of Aruco tags and an error metric was defined that allowed for the estimation of the error in the pose estimates. The result is a substantial improvement in pose estimates when compared with a combination of Aruco tags on a 2D surface. From the benchmark process, it was observed that the 3D grid board led to an average improvement of 40% across all detected parameters related to the pose. We are fully aware that the better calibration of the cameras could lead to better results, and this was also proved in this paper. Regardless of the type of calibration, we acquire better pose estimation with the proposed 3D placement strategy than with the traditional 2D grid. Further work on this topic could be done to determine the best relative orientation between tags themselves for an even better estimate of the pose. More positions and orientations can be added to the benchmark process to make it more robust.

## Figures and Tables

**Table 1:** Parameters of the 2D grid board.

Number of Tags on the Board	2 × 2
Tag edge length	70 mm
Spacing between tags	10 mm
Tag type	6 × 6 × 1000

**Table 2:** Measurement configurations.

Axis/°	−30°	−15°	0°	15°	30°
Roll	YES	YES	YES	YES	NO
Pitch	NO	NO		YES	NO
Yaw	NO	NO		YES	NO

**Table 3:** Results from the benchmark for the 2D Aruco grid board, deviations along each detected parameter of the tags for various positions of the grid board in the camera field of view (FOV).

	Roll	Pitch	Yaw	X	Y	Z
+15° Roll	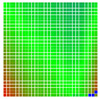	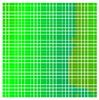	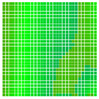	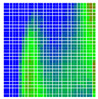	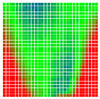	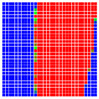
−15° Roll	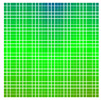	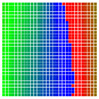	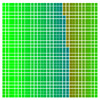	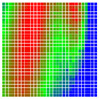	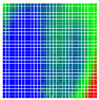	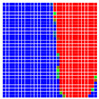
−30° Roll	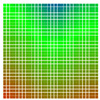	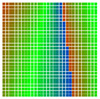	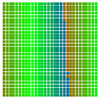	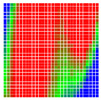	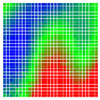	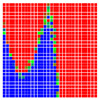
+15° Pitch	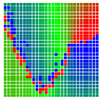	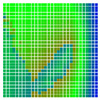	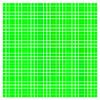	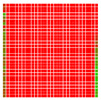	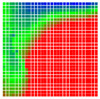	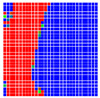
+15° Yaw	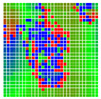	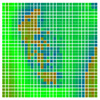	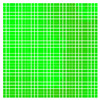	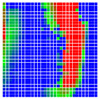	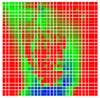	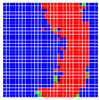
0° (Parallel to the imaging plane)	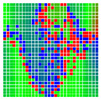	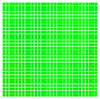	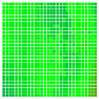	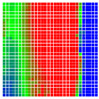	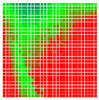	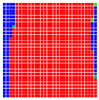

**Table 4:** Histogram of deviations for the 2D grid board pose estimates. The bars further from the center of the bar graph represent the count of measurements with more significant deviations.

	Roll	Pitch	Yaw	X	Y	Z
+15° Roll	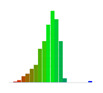	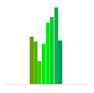	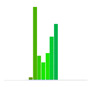	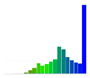	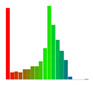	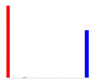
−15° Roll	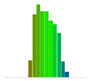	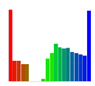	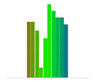	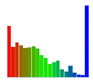	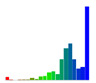	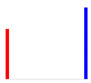
−30° Roll	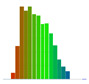	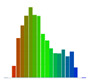	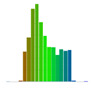	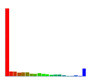	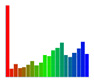	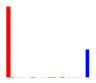
+15° Pitch	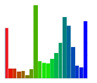	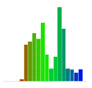	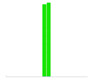	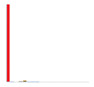	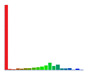	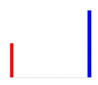
+15° Yaw	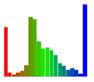	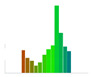	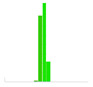	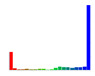	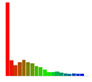	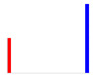
0° (Parallel to the imaging plane)	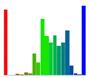	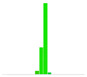	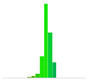	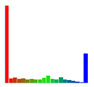	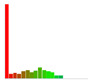	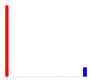

**Table 5:** Three-dimensional grid board parameters.

Number of Tags on the Grid Board	5
Tag edge length	70 mm
Spacing between tags	30 mm
Tag type	6 × 6 × 1000

**Table 6:** Labels for individual measurement results.

	Old Calibration	New Calibration
2D grid board	Measurement 1 (M1)	Measurement 3 (M3)
3D grid board	Measurement 2 (M2)	Measurement 4 (M4)

**Table 7:** Performance comparison between the Aruco grid boards described in this paper. The normal to the tag is parallel with the camera Z-axis.

	Roll	Pitch	Yaw	X	Y	Z
M0	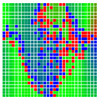	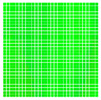	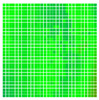	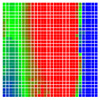	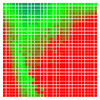	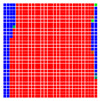
M1	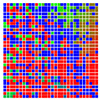	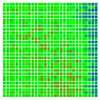	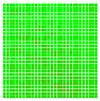	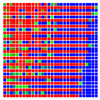	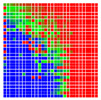	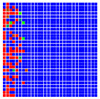
M2	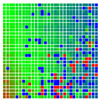	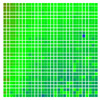	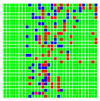	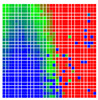	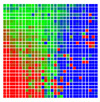	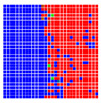
M3	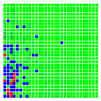	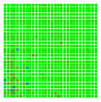	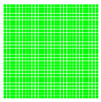	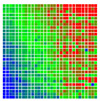	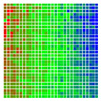	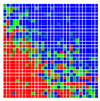
M4	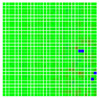	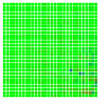	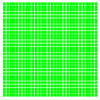	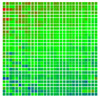	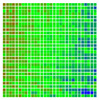	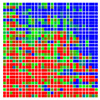

**Table 8:** Histogram of deviations for the measured data. The normal to the tag was parallel with the camera Z-axis. The bars further from the center of the bar graph represent the count of measurements with more significant deviations.

	Roll	Pitch	Yaw	X	Y	Z
M0	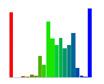	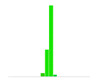	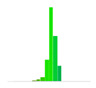	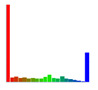	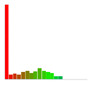	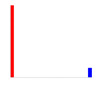
M1	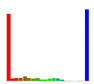	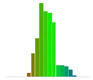	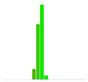	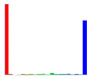	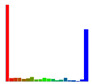	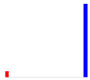
M2	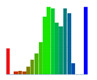	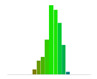	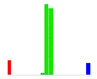	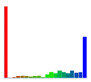	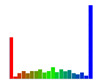	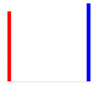
M3	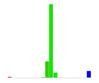	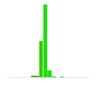	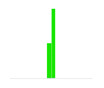	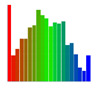	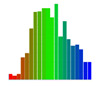	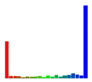
M4	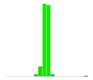	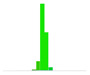	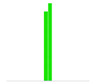	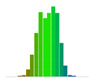	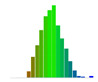	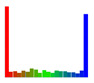
